# The Role of Purinergic Signaling on Deformation Induced Injury and Repair Responses of Alveolar Epithelial Cells

**DOI:** 10.1371/journal.pone.0027469

**Published:** 2011-11-08

**Authors:** Hewan A. Belete, Rolf D. Hubmayr, Shaohua Wang, Raman-Deep Singh

**Affiliations:** Thoracic Diseases Research Unit, Division of Pulmonary and Critical Care Medicine, Mayo Clinic, Rochester, Minnesota, United States of America; Leiden University Medical Center, The Netherlands

## Abstract

Cell wounding is an important driver of the innate immune response of ventilator-injured lungs. We had previously shown that the majority of wounded alveolus resident cells repair and survive deformation induced insults. This is important insofar as wounded and repaired cells may contribute to injurious deformation responses commonly referred to as biotrauma. The central hypothesis of this communication states that extracellular adenosine-5′ triphosphate (ATP) promotes the repair of wounded alveolus resident cells by a P2Y2-Receptor dependent mechanism. Using primary type 1 alveolar epithelial rat cell models subjected to micropuncture injury and/or deforming stress we show that 1) stretch causes a dose dependent increase in cell injury and ATP media concentrations; 2) enzymatic depletion of extracellular ATP reduces the probability of stretch induced wound repair; 3) enriching extracellular ATP concentrations facilitates wound repair; 4) purinergic effects on cell repair are mediated by ATP and not by one of its metabolites; and 5) ATP mediated cell salvage depends at least in part on P2Y2-R activation. While rescuing cells from wounding induced death may seem appealing, it is possible that survivors of membrane wounding become governors of a sustained pro-inflammatory state and thereby perpetuate and worsen organ function in the early stages of lung injury syndromes. Means to uncouple P2Y2-R mediated cytoprotection from P2Y2-R mediated inflammation and to test the preclinical efficacy of such an undertaking deserve to be explored.

## Introduction

Alveolar epithelial plasma membrane [1] wounding is important in the pathogenesis of acute lung injury (ALI) and ventilator induced lung injury (VILI) [2], [3]. The term ‘injury’ has been used to describe diverse biologic stress responses including altered gene and protein expressions, inefficient gas exchange, impaired vascular barrier properties, parenchymal inflammation, fibro-proliferation and microvascular coagulopathy[4], [5], [6]. This report will focus on mechanisms of PM wounding and repair in type I alveolar epithelial cells (type 1 AEC) as one potential determinant of these injury manifestations. Using an *ex vivo* ventilated and perfused rat lung preparation we had previously shown that the majority of wounded alveolus resident cells repair and survive deformation induced insults [7]. This is important insofar as wounded and repaired cells activate stress response genes [8], release pro-inflammatory mediators and may thereby contribute to injurious deformation responses commonly referred to as biotrauma [9].

The central hypothesis of this communication states that extracellular ATP promotes the repair of wounded alveolus resident cells by a P2Y2-R dependent mechanism. This hypothesis is a logical deduction of several well established observations: a) successful PM repair requires the coordinated interplay between exocytic and endocytic membrane trafficking and remodeling events [10], [11], [12]; b) calcium is an essential second messenger in these processes [1]; c) ATP is a secretagogue [13] and is found in abundance in the alveolar exudate of injured lungs [14], [15], [16]; d) airway and alveolar epithelial cells including type 1 AEC express both metabotropic (P2Y) and ionotropic (P2X) purinergic receptors [17], e) stressed cells release ATP, which in turn alters the set point of numerous signal transduction pathways through autocrine activation of ionotropic and metabotropic purinergic receptors [17]; f) there is considerable overlap in the signaling pathways that are activated during cell deformation, PM repair and pathways activated by P2Y-R agonists [1], [13], [18], [19], [20], [21], [22], [23], [24].

Using lung epithelial cell lines as well as primary type 1 AEC rat cell culture models subjected to micropuncture injury and/or deforming stress we show that 1) graded stretch causes a dose dependent increase in cell injury and ATP media concentrations; 2) enzymatic depletion of extracellular ATP reduces the probability of stretch induced PM wound repair; 3) enriching extracellular ATP concentrations facilitates PM wound repair; 4) purinergic effects on cell repair are mediated by ATP and not by one of its metabolites; and 5) ATP mediated cell salvage depends at least in part on P2Y2-R activation. The clinical and biologic implications of these findings for cell repair targeted interventions in lung injury syndromes will be discussed.

## Results

### Percentage of mortally wounded cells and ATP media concentrations vary in a strain- dependent manner

**Figure 1:**
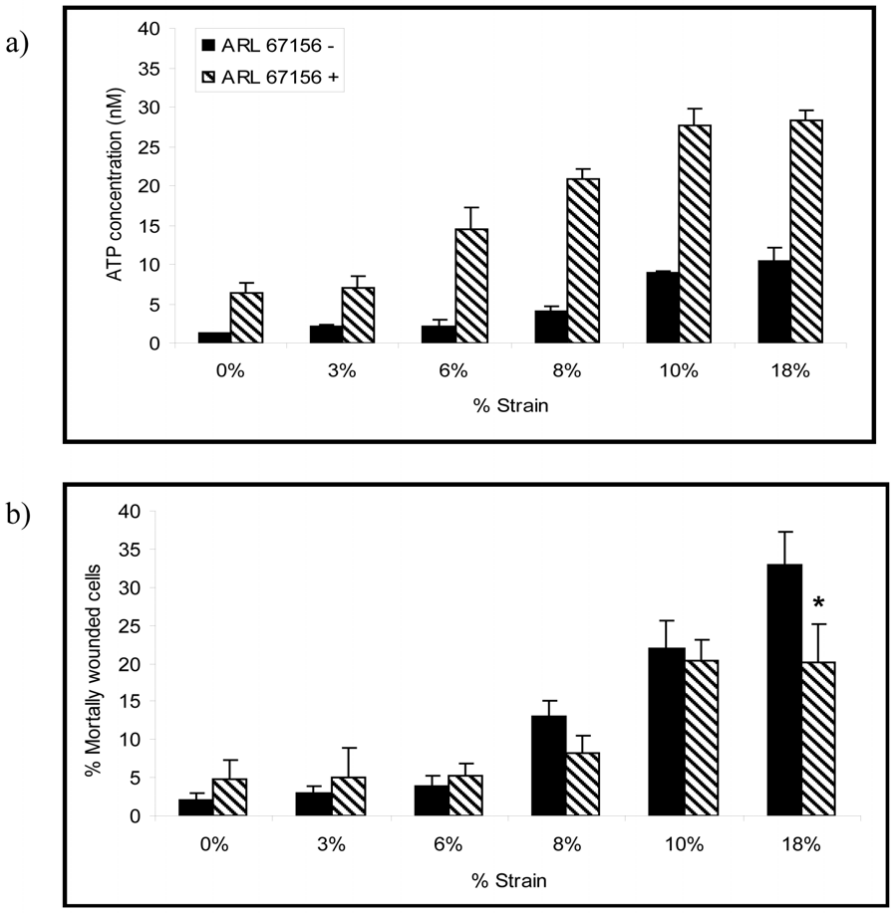
Effect of strain amplitude on medium ATP concentration and % mortally wounded type 1 AECs. Type 1 AECs grown on Flexcell culture plates were stretched in the presence (hatched bars) or absence (black bars) of the ectoenzyme inhibitor ARL 67156 (100 µM) for 10 minutes (frequency = 0.5 Hz and strain rate = 20% sec^−1^). ATP concentration in supernatant was measured using a luciferase assay. (a) Plot of strain amplitude, defined as the % radial length change during each stretch cycle vs. nanomolar ATP media concentration; (b) Plot of strain amplitude vs. % mortally wounded cells as indicated by PI labeling (n = 6). (*  =  p<0.05). Data are presented as means ± one standard error of the mean.

The interactive effects of strain on ATP release and PM integrity were measured in confluent monolayers of type 1 AEC. Strain, defined as the % radial length change of the monolayer during each stretch cycle, was associated with an amplitude dependent increase in ATP media concentration (Figure 1). ATP concentrations were in the nanomolar range and were predictably higher when ATP degradation was prevented by the ectonucleotide pyrophosphatase inhibitor ARL 67156 (hatched bars). A corresponding analysis of cell fate (% mortally wounded cells) suggested a strain injury threshold between 6 and 8% (Figure 1B). Note that the strain at which ATP media concentrations increased compared to baseline averaged between 3% and 6% (Fig. 1A) i.e. was below the strain injury threshold. This indicates that deformation related cellular ATP release does not require PM wounding. Prevention of ATP degradation by ecto-nucleotide pyrophosphatase inhibitor ARL 67156 in samples exposed to injurious strains was associated with fewer mortally wounded cells. This observation is consistent with the postulated ATP mediated cytoprotection. However, the effect was statistically significant only at the highest (most injurious) strains.

### Extracellular ATP facilitates plasma membrane wound repair

**Figure 2:**
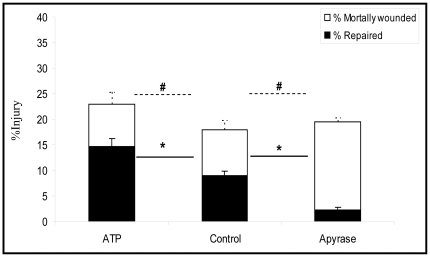
Effects of extracellular ATP and Apyrase on % injury and % repair of type 1 AECs. Apyrase (20 U/ml) treated, ATP (10 µM) treated and control cells were subjected to cyclic stretch of 10% for 10 minutes (Frequency = 0.5 Hz strain rate = 20% sec^−1^) in the presence of FDx. PI labeling was used to identify mortally wounded cells. % injured, % repaired and % mortally wounded rates were calculated according to the formula described in the methods section. The y axis represents the percentage of cells that were injured as a result of stretch (% injury). The white bar represents cells that were injured & repaired, while the black bar represents mortally wounded cells, i.e. cells that were injured but failed to repair (n = 6) (*  =  p<0.05, # p>0.05). Data are presented as means ± one standard error of the mean.

Type 1 AEC monolayers were exposed to injurious deformations (10% strain for 10 minutes) while ATP media concentrations (and by inference ATP concentrations at the PM) were experimentally manipulated (Figure 2). The enzymatic degradation of ATP subsequent to its deformation induced release was associated with a dramatic reduction in the rate of PM wound repair. While under control conditions 51±3 % of injured cells successfully resealed PM wounds, in the absence of extracellular ATP this fraction fell to 12±2% (p<0,01). In comparison the addition of exogenous ATP (10 µM) increased the rate of PM wound repair from 51±3 to 64±3% (P<0.05). While the availability of extracellular ATP clearly influenced cell fate (see subdivisions within the columns of Figure 2), manipulating the ATP media concentrations had no effect on the probability of stretch induced cell injury, specifically on the probability of PM stress failure.

### Cytoprotection is mediated by ATP and not by one of its metabolites

**Figure 3:**
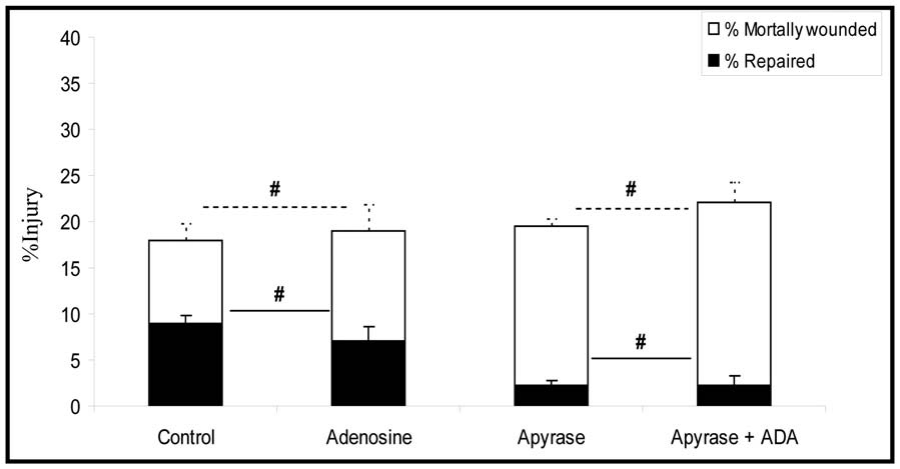
Effects of adenosine and adenosine deaminase on % injury and % repair of type 1 AECs. Adenosine treated, adenosine deaminase + apyrase treated and control cells were subjected to cyclic stretch of 10% for 10 minutes (Frequency = 0.5 Hz strain rate = 20% sec^−1^) in the presence of FDx. PI labeling was used to identify mortally wounded cells. % injured, % repaired and % mortally wounded rates were calculated according to the formula described in the methods section (n = 6) (# p>0.05) Data are presented as means ± one standard error of the mean.

To determine if adenosine, which is generated during AMP hydrolysis, modulates ATP dependent effects on cell repair, type 1 AEC were stretch-injured while media concentrations of adenosine were manipulated. The addition of 5 µg/ml adenosine to the media of stretch wounded cells had no significant effect on the rates of cell repair which averaged 37±8% and 51±3% respectively; (p>0.05). Similarly, preparations treated with Apyrase, had the same low repair rates regardless of whether endogenously generated Adenosine was degraded with adenosine deaminase (ADA 5 U/ml) or not (Figure 3).

### Alveolar epithelial cell lines and primary rat Type 1 AEC's express P2Y2 purinergic receptors in culture

**Figure 4:**
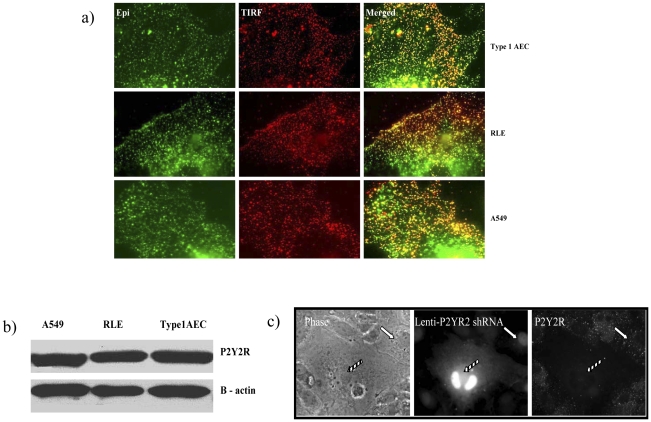
P2Y2-R expression in type 1 AEC, A549 and RLE cells. (a) Representative Epi and TIRF images of type 1 AECs, A549 and RLE immunostained with anti-P2Y2-R. TIRF mode (red) and EPI mode [70] images were overlaid (yellow) to show colocalization. (b) Protein abundance of P2Y2-R (top) in relation to loading control, beta actin (bottom), in type 1 AEC, A549 and RLE cells. (c) Immunostaining showing P2Y2-R null (gridded arrows) and P2Y2-R positive (white arrow) type 1 AEC. Left panel shows phase images of type 1 AEC culture; middle panel shows GFP fluorescence as a result of lenti-P2Y2-R shRNA infection; right panel shows expression of P2Y2-R. Note the absence of P2Y2-R immunolabel in the null cell (gridded arrow) compared to the surrounding uninfected wild type control cells.

P2Y2-R is a Gq11-coupled protein that is activated equipotently by extracellular ATP and UTP, leading to Gq-dependent activation of Phospholipase C (PLC), which in turn regulates calcium flux and Protein kinase C (PKC) activity by an Inositol trisphosphate (IP3) and diacylglycerol (DAG) dependent mechanism [25]. P2Y2-R is up regulated in response to stress and has been implicated in epithelial migration and wound repair (distinct from repair of PM wounds in single cells) [26]. In order to test the hypothesis that ATP's actions on cell repair are mediated via P2Y2-R activation, we needed to demonstrate that this receptor is expressed at the PM in our culture models. This was indeed the case in all three models (type 1 AEC, RLE, A549) as shown by the colocalization of P2Y2-R immunolabels in total internal reflection fluorescence (TIRF) and epifluorescence (Epi) mode images (Figure 4A) as well as by Western blotting (Figure 4B). Differences in immunolabeling between primary Type 1 AEC's in which P2Y2-R was knocked down (shRNA) using a lentiviral approach and in uninfected wild type cells established the specificity of the P2Y2-R immunolabel (Fig. 4C).

### P2Y2 purinergic receptor silencing and knockdown is associated with low rates of PM wound repair

**Figure 5:**
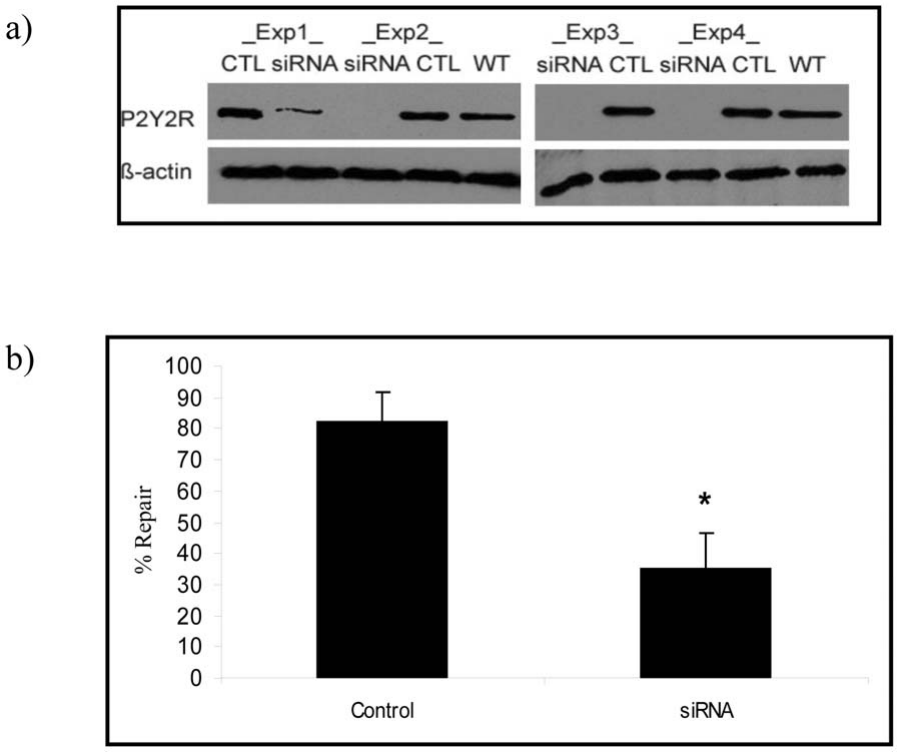
Effect of P2Y2-R silencing (via siRNA) on PM wound repair in A549 cells. (a) P2Y2R protein expression in P2Y2R-targeted siRNA transfected A549 cells (siRNA) compared to those transfected with scrambled control (CTL) and wild-type A549 (WT) (top). Beta-actin was used as a loading control (bottom). Western blots shown are results of four separate experiments (Exp1-4) (b) A549 cells transfected with P2Y2R-targeted siRNA (siRNA) or non-targeting scrambled control siRNA (control) were subjected to micropuncture injury. % repair was calculated according to the formula described in the methods section (n = 17 cells/ group) (* indicates p<0.05). Data are presented as means ± one standard error of the mean.

**Figure 6:**
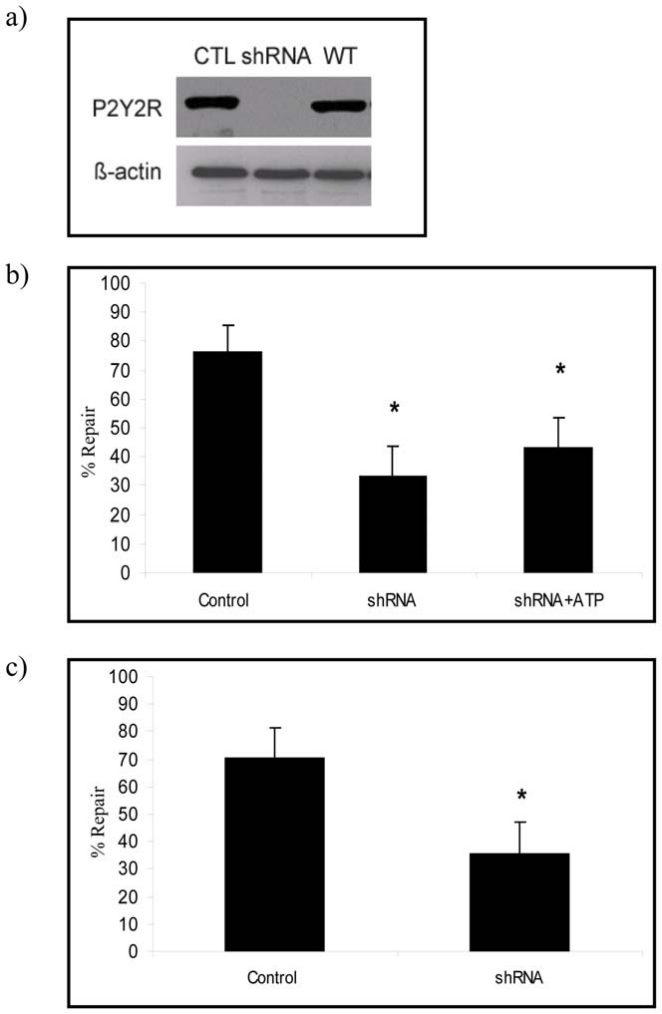
Effect of P2Y2-R knockdown (via shRNA) on PM wound repair in RLE and Type 1 AECs. (a) P2Y2R protein expression in P2Y2R-shRNA lentivirus infected RLE cells (shRNA) compared to those that were infected with non-targeting shRNA lentiviral particles (CTL) and wild type RLE cells (WT) (top). Beta-actin was used as a loading control (bottom). (b) P2Y2R null (shRNA) and P2Y2R positive RLE (control) were subjected to micropuncture injury in the presence or absence of ATP and % repair was calculated according to the formula described in the methods section (n = 21 cells/group). (c) P2Y2R null (gridded arrows) and P2Y2R positive (white arrow) type 1 AECs were subjected to micropuncture injury and % repair was calculated according to the formula described in the methods section (n = 17 cells/ group) (* indicates p<0.05). Data are presented as means ± one standard error of the mean.

To determine if ATP mediated enhancement of cell repair requires P2Y2–R activation, the fate of micropuncture-injured P2Y2-R null cells was compared with that of injured wild type controls. In all instances in which P2Y2-R protein expression of A549 cells could be reduced by gene silencing, (Figure 5A) the number of wounded cells with successful membrane repair was significantly lower than that of injured wild type controls (Figure 5B). The same was true for micropuncture-injured RLE and primary type 1 AEC's in which gene and protein expressions were knocked down using a lentiviral approach (Figures 6 and 4C). Moreover, the addition of ATP to media failed to restore the repair rates of P2Y2-R −/− cells to normal values (Figure 6B).

### Lysosomal-Associated Membrane Protein 1 (LAMP1) exocytosis is under purinergic control

**Figure 7:**
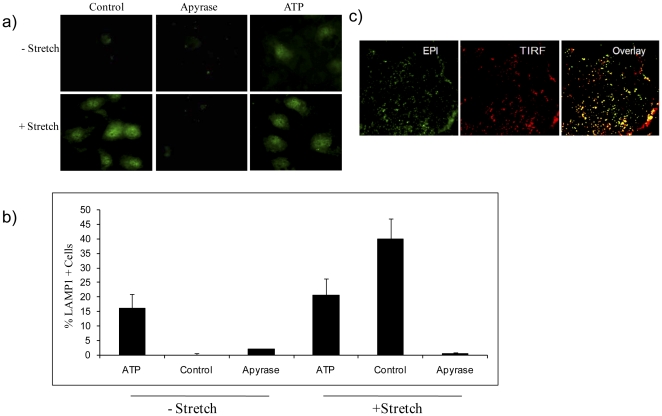
Surface LAMP1 expression in live type 1 AECs. (a) Epifluorescence images of LAMP1 immunostaining in type 1 AEC. Cells were treated with 10 µM of ATP or 20 U/ml Apyrase in the presence or absence of stretch (amplitude = 6% frequency = 0.5 Hz strain rate = 20%sec^−1^ t = 8 min) and were labeled with LAMP-1 antibody at 4°C. (b) Percentages of LAMP1 positive cells were quantified by dividing the number of LAMP1 positive cells by the total number of cells in each image field. Data are presented as means ± one standard error of the mean. (c) TIRF (red) and EPI [70] fluorescence images were overlaid (yellow) to validate the expression of LAMP1 at the PM.

Successful repair of PM lesions requires the fusion of calcium sensitive endo-membranes, generally of lysosomes, with the PM in the vicinity of the wound [27]. In an attempt to establish mechanistic interactions between deformation induced ATP release and the consequent autocrine stimulation of lysosomal exocytosis, primary type 1 AEC's were exposed to non-injurious strains (6% for 10 minutes) and surface expressions of LAMP-1 was measured. Lamp-1 is an abundant lysosomal membrane glycoprotein, whose NH2-terminal luminal domain becomes exposed at the PM following membrane fusion. As such the molecule can be immuno-labeled and serves as a surrogate marker of lysosomal secretion. In unstressed primary rat type 1 AEC's, the constitutive expression of LAMP-1 at the PM was rather low, but it was significantly increased in response to stretch or the supplementation of media with 10 µM ATP (Fig. 7). The enzymatic degradation of ATP with Apyrase abolished the stretch induced LAMP-1 expression at the PM, thereby establishing ATP as an autocrine inducer of deformation induced lysosomal exocytosis.

To garner additional support for the hypothesis that deformation induced lysosomal exocytosis is regulated in a P2Y2-R dependent manner, the LAMP-1 expressions of cyclically stretched and/or ATP stimulated wild type and P2Y2-R −/− RLE cells were compared. As postulated, following either stimulus P2Y2-R deficient RLE cells expressed significantly less LAMP-1 at the plasma membrane than the corresponding wild type controls.

## Discussion

Our results illustrate a previously unidentified link between mechanotransduction, purinergic signaling and lysosomal exocytosis as important mechanisms of alveolar epithelial PM repair. Both injurious and non-injurious deformations caused type 1 AEC to release ATP (Figure 1A). While extracellular ATP did not alter the probability of stretch induced PM wounding, autocrine ATP release was associated with enhanced PM wound repair (Figs. 1B and 2). This cytoprotective effect was mediated by ATP as opposed to one of its metabolites, and presumably reflects P2Y2 receptor dependent lysosomal exocytosis.

Cell wounding is an important driver of the innate immune response of ventilator-injured lungs [2], [3]. Type 1 alveolar epithelial cell wounding is prevalent in ventilator-injured lungs and contributes to the pathogenesis of biotrauma [28]. Biotrauma is an inflammatory response of the lung parenchyma to mechanical stress, to which not only necrotic cells but also wounded and repaired cells contribute [29], [30]. Processes that are critical for cell repair, such as the trafficking of calcium sensitive endomembranes are also regulated by purinergic input [31], [32]. In fact, there is a great overlap between purinergic receptor activation, stress signaling and mechano-transduction pathways [33], [34], [35], [36]. The functional significance of purinergic signaling in the lung is complex due to the promiscuity of ATP release, involvement of various ecto-enzymes, cell specific distribution of purinergic receptors and availability of the different nucleotides and their respective metabolites [25], [37], [38], [39], [40], [41]. In fact, many lung injury manifestations captured under the term biotrauma could be downstream consequences of purinergic signaling [42], [43]. Therefore, defining the contribution of purinergic signaling to cell repair and understanding whether and how wounded lung cells repair is of interest. This understanding is a prerequisite in the search for cytoprotective interventions in the management of patients with acute lung injury.

We have shown that injurious as well as non-injurious stretch causes the release of ATP from type 1 AECs *in vitro*. While we did not dissect the strain amplitude specific mechanisms of ATP release, we did observe LAMP1 expression, a surrogate of lysosomal exocytosis, at strains as small as 6% (Figure 7). Since ATP is a lysosomal cargo [44], we conclude that lysosomal exocytosis contributed to the strain dependent increase in ATP media concentration. This conclusion is in keeping with prior reports that the deforming stress associated with a media change is a sufficient stimulus for ATP release in cell culture [45], [46], [47]. In a wounded cell cytosolic ATP leaks into the extracellular environment, which probably accounts for the increased rate of ATP release at strain amplitudes ≥8% (Figure 1A). However, lysosomes are a likely source of extracellular ATP in wounded cells as well, since PM wounding triggers the fusion and exocytosis of calcium sensitive endomembranes, including lysosomes [27], [48], [49].

In order to test the hypothesis that extracellular ATP is an important facilitator of PM wound repair, we manipulated both ligand and the receptor of interest, namely P2Y2-R. In initial experiments we inhibited the activity of cell surface resident ectoenzymes, which regulate the concentration of ATP relative to that of its metabolites ADP, AMP, and Adenosine. These enzymes include the ecto-phosphodiesterase/nucleotide pyrophosphatase (E-NPP) family, the ecto-nucleotidase 5′-triphosphate diphosphohydrolase (NTPDase) family, Ecto 5′-nucleotidase/CD73 and alkaline phosphatases [38]. Inhibition of ATP degradation, following its stretch induced release, by the selective ecto-ATPase inhibitor ARL 67156 provided the initial clue that our hypothesis would be accepted. Significantly fewer cells were mortally wounded when AEC's were stretched by 18% in the presence of ARL 67156. (Figure 1B) Since AEC's typically repair PM lesions within 20 seconds [10] we interpreted cells that labeled PI (propidium iodide) positive 2 minutes after an insult as mortally wounded, i.e. necrotic. Subsequent experiments established that the cytoprotective effect of ARL 67156 was attributable to an increase in extracellular ATP concentration, since enriching media with 10 µM ATP reproduced the cytoprotective effects of pharmacologic ectoenzyme inhibition, while depletion of autocrine released ATP with Apyrase was associated with an increase in the number of mortally wounded cells (Figure 2). This conclusion was further amplified by the lack of effect of Adenosine supplementation or its degradation by Adenosine deaminase on cellular repair responses (Figure 3).

We had previously postulated that deformation induced lipid trafficking (DILT) was an important cytoprotective mechanism in the prevention of PM stress failure [43]. Therefore, we sought to test if ATP, which is a secretagogue for type II AEC's, prevented PM wounding distinct from promoting wound repair. To this end we repeated cell deformation experiments in presence of fluorescein isothiocyanate Dextran (FDx), which in a wounded cell gains access to the cytosol and gets trapped there provided the PM lesion reseals. However, using this approach we could not demonstrate a cyclic nucleotide mediated cytoprotection. In other words, enriching the ATP concentration at the PM of type I AEC's did not alter their probability of deformation induced wounding (Figure 2), leading us to conclude that the primary cytoprotective mechanism of ATP on the alveolar epithelium is wound repair and not injury prevention. As suggested by our previous work [42], [43] we did observe a DILT response even at non-injurious strains, and based on LAMP-1 PM expression now show that DILT involves lysosomal exocytosis (Figure 7). Since enzymatic degradation of ATP resulted in a dramatic reduction of LAMP-1 surface expression in stretched AEC's, our data also suggest that DILT is an autocrine ATP mediated purinergic deformation response which does not require a PM defect.

**Figure 8:**
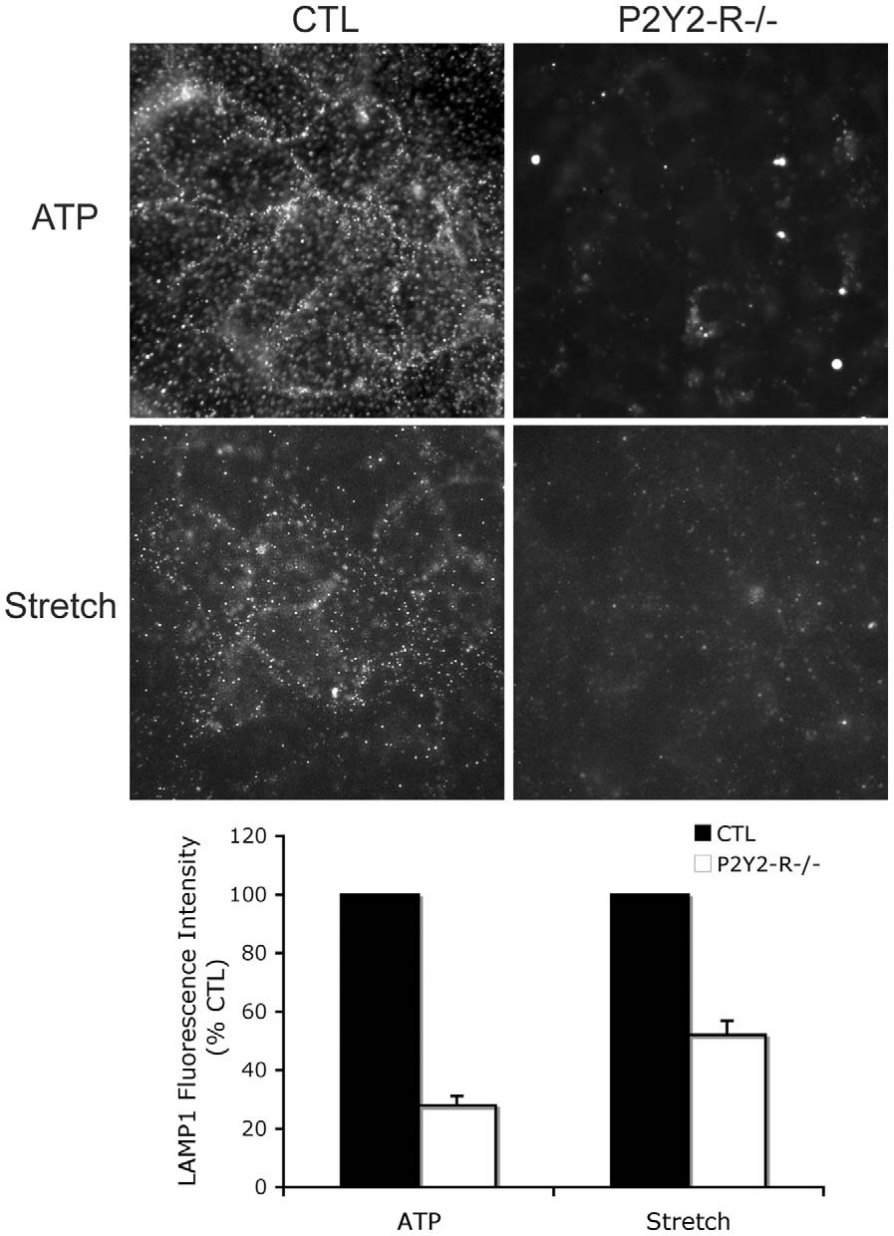
Plasma membrane LAMP1 in wild type (CTL) and P2Y2R −/− RLE cells. Cells were either treated with 50 µM ATP (top row, n = 5) or stretched (middle row; amplitude = 10% frequency = 0.5Hz strain rate = 40%sec^−1^ t = 8 min, n = 4) and subsequently labeled with LAMP-1 antibody at 4°C. Fluorescence intensity of P2Y2-R −/− RLE cells was expressed as % of the corresponding wild type CTL and data presented as means ± one standard error of the mean (bottom row).

Having identified ATP as the responsible ligand, we next asked which of the P2 family of purinergic receptors was most likely to regulate processes associated with lysosomal exocytosis and cell repair. P2Y2-R is a Gq11-coupled protein that is activated equipotently by extracellular ATP and UTP, leading to Gq-dependent activation of PLC, which in turn regulates calcium flux and PKC activity by an IP3 and DAG dependent mechanism [50], [51]. These pathways are also involved in the regulation of membrane trafficking and intersect with PKA (Protein kinase A) and cAMP (cyclic adenosine monophosphate) stimulated secretion [52], [53]. P2Y2-R is upregulated in response to stress and has been implicated in epithelial migration and wound repair, distinct from repair of PM wounds in individual cells [26], [27], [54]. In order to test if ATP's role in facilitating cell repair was regulated by P2Y2-R, we needed to demonstrate that primary type 1 ACE's expressed this protein at the time of cell harvest and retained its expression after days in liquid culture. We did so using immunostaining and immunoblotting approaches and subsequently showed that primary AEC's in which the gene was “knocked down” no longer expressed the protein at the PM (Figures 4 and 6). This, in turn, allowed us to test and ultimately demonstrate that P2Y2-R negative cells were deficient in ATP and stretch induced lysosomal exocytosis (Figure 8) and less likely to repair micropuncture wounds than wild type controls (Figures 5 and 6).

Our studies of the purinergic control of cell repair were conducted on alveolar epithelial monolayers exposed to injurious stress. Cells were wounded either by micropuncture or by stretch. The relatively short duration of the experimental stress exposure and the interval following which stress responses were analyzed was dictated by plasma membrane wound repair time, which is typically on the order of tens of seconds. Notwithstanding the strong rationale linking cell wounding and inflammation, inferences about the long term consequences of deforming stress on lung structure and function are hypothetical. While the abundance of ATP in alveolar exudates and the existence of alveolar epithelial wounds in ventilator injured lungs of animals and patients is well established [7], [14], [55], questions about the *in situ* wounding mechanisms and specific strain injury thresholds remain largely unresolved [56]. Nevertheless, we note that the in vitro deformation amplitudes we have employed fall within the physiologic range of morphometric alveolar wall strain [57] and suggest that the mechanisms we have detailed in a reduced system operate in the intact alveolus as well. We have focused on the P2Y2-Receptor because it is more sensitive to ATP and UTP than their metabolites and because P2Y2-R signaling intersects cellular mechanotransduction and stress pathways at multiple levels [58], [59]. It is therefore not surprising that at least in reduced systems many of the clinical and cell biologic manifestations of “injurious” ventilation, such as inflammation, apoptosis, cell growth and migration as well as vesicular exocytosis can be reproduced by P2Y2-R activation in the absence of deforming stress.

Our estimates of the dose response relationships between deforming stress, ATP release and P2Y2–R activation dependent deformation responses were based on changes in ATP media concentrations (Figure 1B). In this context, it should be noted that ATP media concentrations are typically in the nano-molar range, even though P2Y2-R activation is thought to require micromolar quantities of ATP. This discrepancy was recently addressed by Okada *et al*. who used a cell based bioluminescence assay to show that the ATP concentration at the surface of hypotonically stressed airway epithelial cells was several orders of magnitude greater than that in media [60]. In other words, released ATP is not only readily metabolized by ectoenzymes, but also greatly diluted in liquid media.

Acceptance of our hypothesis, i.e. demonstration of the proposed ATP effect in a reduced alveolar injury model, warrants a discussion of potential risks and benefits of targeting P2Y2-R activation and/or purinergic signaling in preclinical injury models and ultimately patients. ATP mediated cell salvage therapy, while in principle attractive, may be accompanied by a pro-inflammatory response, which in the short term would be detrimental for gas exchange and would likely be interpreted as aggregated tissue damage. Purinergic proinflammatory mechanisms have been linked to P2Y as well as P2X7 receptor activation [61], [62], [63]. Moreover wounded and repaired alveolus resident cells could serve as governors of a coordinated injury response by uninjured neighbors. There is growing evidence that paracrine purinergic signals regulate cross compartmental calcium oscillations in pulmonary microvessels and that gap junction communications between alveolus resident epithelial and endothelial cells represent pathways for mechanotransduction and surfactant release [14], [64]. In line with this hypothesis the endotracheal instillation of ATP in a murine VILI model was associated with an increase in permeability edema and an influx of inflammatory cells [65]. Conversely, purinergic receptor-deficient (P2Y1/P2Y2−/−) mice were shown to have impaired inflammatory responses and unable to clear *Pseudomonas aeroginosa* from the lung [66]. Underscoring the complexity of purine mediated lung responses the intravenous administration of a degradation resistant ATP analogue, ATPγS exerted a protective role against endotoxin induced lung injury by preserving the integrity of endothelial cell-cell junctions.

In summary, our observations underscore the critical yet multifaceted role of ATP in shaping lung parenchymal stress responses. Although our research has primarily focused on one specific ATP/ P2Y2-R mediated effect, namely cell repair, for the clinician and systems biologist the data pose a challenge of having to place this specific effect into the broader context of alveolar inflammation, tissue remodeling and lung repair. While rescuing cells from wounding induced death may seem appealing, it is quite possible that survivors of membrane wounding become governors of a sustained pro-inflammatory state and thereby perpetuate and worsen organ function in the early stages of the syndrome. Means to uncouple P2Y2-R mediated cytoprotection from P2Y2-R mediated inflammation and to test the preclinical efficacy of such an undertaking deserve to be explored.

## Materials and Methods

*Note:* All materials were obtained from Sigma-Aldrich (St. Louis, MO) unless otherwise noted. Abbreviations are defined in Table 1.

**Table 1:** Key to abbreviations used in the text.

Abbreviations	Meaning
ALI	Acute Lung Injury
VILI	Ventilator Induced Lung Injury
AEC	Alveolar Epithelial Cell
ATP	Adenosine-5′-triphosphate
ADP	Adenosine diphosphate
AMP	Adenosine monophosphate
UTP	Uridine-5′-triphosphate
LAMP1	Lysosomal-associated membrane protein 1
PM	Plasma membrane
PI	Propidium iodide
FDx	Fluorescein isothiocyanate–dextran
ADA	Adenosine deaminase
E-NPP	ecto-phosphodiesterase/nucleotide pyrophosphatase
NTPDase	ecto-nucleotidase 5′-triphosphate diphosphohydrolase
ARL 67156	6-*N*,*N*-Diethyl-D-β,γ-dibromomethyleneATP trisodium salt
DILT	deformation induced lipid trafficking
PKC	Protein kinase C
PKA	Protein kinase A
IP3	Inositol trisphosphate
DAG	diacylglycerol
cAMP	Cyclic adenosine monophosphate

### Cell Culture

Human lung adenocarcinoma epithelial cell line (A549) and Rat Lung Epithelial Cell line [4] were obtained from ATCC (Manassas, VA**)**. A549 and RLE cells were cultured either on a 35 mm glass bottom dishes (MatTek, Ashland, MA) or on collagen coated six-well flexcell culture plates (Flexcell International, Mc-Keesport, PA) in F12K growth medium (Invitrogen, Carlsbad, CA) supplemented with 10% fetal bovine serum,100 units/ml penicillin and 100 µg/ml streptomycin and incubated at 5% CO_2_ at 37°C. Type 1 AEC were isolated from rat lungs as recently described [67] and were cultured to confluence on a 35 mm glass bottom dishes or on collagen coated six-well flexcell culture plates in Dulbecco's Modified Eagle Medium (DMEM) (Invitrogen, Carlsbad, CA) supplemented with 10% fetal bovine serum, 100 units/ml penicillin and 100 µg/ml streptomycin and incubated at 5% CO_2_ at 37°C. The type 1 AEC isolation protocol was approved by the Institutional Animal Care and Use Committee (IACUC) of the Mayo Clinic (IACUC number A15210).

### Stretch Assay

A detailed description of the method and validation of the stretch assay can be found in Belete et al and Stroetz et al [68], [69]. Cells grown on collagen coated flexcell membranes were stretched in the presence of 0.25% fluorescein isothiocyanate Dextran (FDx) and were allowed to repair for 2 minutes before unbound dye was rinsed off. Fluorescence images were obtained at emission peaks of 510 and 620 nm, using an inverted Axiovert 200 M microscope equipped with 40X water lens (Carl Zeiss, Thornwood, NY).

### Assessment of cell wounding and repair

Cell wounding and repair were assessed using our previously published dual labeling method [68], [69]. Green FDx positive cells are considered wounded and healed because restoration of membrane integrity has trapped FDx in their cytosol. In contrast, PI positive cells with red nuclear fluorescence are considered mortally wounded because failure to reseal the membrane defect within minutes precludes subsequent repair.

### Injury and repair percentage calculations

The relative fractions of repaired and mortally wounded cells was calculated by dividing the number of green and red cells by the total number of injured cells (red + green) respectively. The fraction of injured cells was calculated by dividing the number of injured cells (red + green) by the total number of cells per image field. Note that injured cells are cells that are injured as a result of mechanical deformation irrespective of whether they were able to repair or not.

### Micropuncture Assay

A detailed description of the method and validation of micropuncture wounding assay can be found in Belete et al. [68], [69]. In short, a motorized microinjector (InjectMan; Eppendorf, Hauppauge, NY) was used to position a 1 µm diameter glass needle (Femtotip®, Eppendorf) above the targeted cell and the cell was punctured at a 45° angle with for 0.3 seconds. Successful wound repair was verified by nuclear exclusion of PI (4 µl/ml). Hence, % repair was calculated as [1 – (# PI positive cells/ total micropunctured cells) * 100%].

### ATP concentration measurements

Type 1 AEC monolayers grown on collagen coated six-well flexcell culture plates were rinsed and the culture medium was replaced with serum-free Earle's medium with or without 100 µM ARL-67156 (to inhibit extracellular ATP degradation by ectoenzymes). 100 µl aliquot of medium was harvested, quick frozen, and stored at –20°C. The cells were then stretched and a second 100-µl aliquot of medium was harvested and quick frozen until ready for use. ATP concentration was measured in both pre and post stretch medium with the ATP Bioluminescent Assay kit according to the manufacturer's instructions (Sigma-Aldrich, St. Louis, MO).

### P2Y2-R Immunostaining

Fixed cells were incubated with rabbit anti-P2Y2-R followed by Alexa fluor 488 secondary antibody. Epi and TIRF images were obtained at an emission peak of 510 nm, using IX70 microscope equipped with Olympus TIRF module using 100X TIRF lens with 1.45NA.

### Live cell lamp 1 labeling

Type 1 AEC monolayers were stretched at 37°C & 5% CO_2_ for 8 minutes (Amplitude = 6% Frequency = 0.34 Hz & velocity = 20% ^−1^sec). Cells were immediately washed with ice cold HMEM (Minimum Essential Medium Eagle Modified with Hanks' Salts) and incubated with anti-LAMP-1-H-228 on ice for 30 minutes (rabbit polyclonal antibody raised against amino acids 1–228 of LAMP-1, Santa cruz, CA). Cells were washed with cold HMEM, fixed with 2% formaldehyde and incubated with of Alexa fluor 594 goat anti-rabbit secondary antibody for 30 minutes. Epifluorescence images were obtained at an emission peak of 620 nm, using Olympus AX70 microscope equipped with 40X water lens with 0.6 NA.

### Western Blot

Cells were homogenized in the presence of protease inhibitor cocktail and centrifuged at 10,000 x *g*. The pellet was resuspended in sample buffer, and separated by SDS-PAGE. Proteins were electro-transferred onto a nitrocellulose membrane. The membranes were incubated with rabbit anti-P2Y2-R or mouse anti-Beta actin primary antibodies followed by goat anti-rabbit or anti-mouse horseradish peroxidase-conjugated secondary antibodies (CalBiochem, San Diego, CA). Proteins of interest were visualized using the enhanced chemiluminescence detection system (Thermo Scientific, Rockford, IL).

### siRNA Transfection

24 hour prior to transfection, 1×10^5^ A549 cells were plated on a 6 well plate in antibiotic free growth medium. After 24 hour incubation, RNA mixtures were added. The RNA mixtures were prepared as follows: 10 nM of P2Y2-R SiRNA (Ambion ID s9966, Austin, TX) or 10 nM of non-targeting scrambled control siRNA (Ambion ID AM4621, Austin, TX) were diluted in 100 µl Opti-MEM Reduced Serum Medium (Invitrogen, Carlsbad, CA). 5 µl/well of Lipofectamine 2000 (Invitrogen, Carlsbad, CA) was also diluted in 100 µl Opti-MEM. The siRNA Lipofectamine 2000 were mixed and incubated for 20 minutes at room temperature. 200 µl of SiRNA-Lipofectamine 2000 complex was added to each well and incubated at 37°C for 24 hours. After 24 hours the media was changed and cells were further grown for 72–96 hours before western blot lysates were collected.

### shRNA Transfection

100 uL of bacterial glycerol stocks of human GIPZ lentiviral P2Y2-R shRNA (Open biosystems, Huntsville, AL) and control shRNA were mixed with 5 ml of LB media with antibiotics. The mixtures were incubated at 37°C for 4 hours (with shaking). The starter cultures were then transferred into LB broth of 250 ml and shaken overnight. After 24 hours, the cells were harvested by centrifuging at 4000 rpm for 20 min at 4 degrees. Qiagen Maxiprep protocol was then carried out according to the manufactures protocol (Qiagen, Valencia, CA). A standard Lipofectamine plasmid DNA transfection was performed on 293 cells (E1-transformed human embryonic kidney cells) according to manufacture's manual. Transfections and viral productions were monitored by GFP expression. Viruses were collected 48–72 hours post transfection and transduction was carried out according to the following protocol. On day zero, 1.6×10^5^ of RLE cells or type 1 AEC were plated on a 6 well plate in complete growth medium. The following day, the growth media was removed and the culture was washed with PBS before 1 uL of 1000x polybrene (Sigma, S-2667) and 1 ml of virus were added to each well. 4–6 hours post-transduction, an additional 1 ml of full media was added to the cells. At 48 hours post-transduction the cells were examined for the presence of reporter expression (GFP) and the efficiency of transduction was determined to be 95%. Puromycin selection was performed on every generation of RLE cells. Immunostaining and western blot experiments were carried out at least 120 hours post-transduction.

### Statistics

Cell injury percentages and ATP concentration measurements are presented as means and standard errors. Binary calculations were used to determine the percentage of repair mean and standard error of means in the micropuncture experiments. All other statistical comparisons were made using analysis of variance (ANOVA). Statistical significance was assumed at p<0.05 with respect to a two-tailed probability distribution.

## References

[1] Shen SS, Tucker WC, Chapman ER, Steinhardt RA (2005). Molecular regulation of membrane resealing in 3T3 fibroblasts.. J Biol Chem.

[2] Dreyfuss D, Saumon G (1998). Ventilator-induced lung injury: lessons from experimental studies.. Am J Respir Crit Care Med.

[3] Vlahakis NE, Hubmayr RD (2005). Cellular stress failure in ventilator-injured lungs.. Am J Respir Crit Care Med.

[4] Gattinoni L, Carlesso E, Cressoni M (2011). Assessing gas exchange in acute lung injury/acute respiratory distress syndrome: diagnostic techniques and prognostic relevance.. Curr Opin Crit Care.

[5] Wheeler AP, Bernard GR (2007). Acute lung injury and the acute respiratory distress syndrome: a clinical review.. Lancet.

[6] Nieuwenhuizen L, de Groot PG, Grutters JC, Biesma DH (2009). A review of pulmonary coagulopathy in acute lung injury, acute respiratory distress syndrome and pneumonia.. Eur J Haematol.

[7] Gajic O, Lee J, Doerr CH, Berrios JC, Myers JL (2003). Ventilator-induced cell wounding and repair in the intact lung.. Am J Respir Crit Care Med.

[8] Grembowicz KP, Sprague D, McNeil PL (1999). Temporary disruption of the plasma membrane is required for c-fos expression in response to mechanical stress.. Mol Biol Cell.

[9] Tremblay LN, Slutsky AS (1998). Ventilator-induced injury: from barotrauma to biotrauma.. Proc Assoc Am Physicians.

[10] Godin LM, Vergen J, Prakash YS, Pagano RE, Hubmayr RD (2011). Spatiotemporal Dynamics of Actin Remodeling and Endomembrane Trafficking in Alveolar Epithelial Type I Cell Wound Healing..

[11] McNeil PL, Steinhardt RA (2003). Plasma membrane disruption: repair, prevention, adaptation.. Annu Rev Cell Dev Biol.

[12] Tam C, Idone V, Devlin C, Fernandes MC, Flannery A (2010). Exocytosis of acid sphingomyelinase by wounded cells promotes endocytosis and plasma membrane repair.. J Cell Biol.

[13] Jung SR, Kim MH, Hille B, Nguyen TD, Koh DS (2004). Regulation of exocytosis by purinergic receptors in pancreatic duct epithelial cells.. Am J Physiol Cell Physiol.

[14] Patel AS, Reigada D, Mitchell CH, Bates SR, Margulies SS (2005). Paracrine stimulation of surfactant secretion by extracellular ATP in response to mechanical deformation.. Am J Physiol Lung Cell Mol Physiol.

[15] Ahmad S, Ahmad A, McConville G, Schneider BK, Allen CB (2005). Lung epithelial cells release ATP during ozone exposure: signaling for cell survival.. Free Radic Biol Med.

[16] Douillet CD, Robinson WP, Zarzaur BL, Lazarowski ER, Boucher RC (2005). Mechanical ventilation alters airway nucleotides and purinoceptors in lung and extrapulmonary organs.. Am J Respir Cell Mol Biol.

[17] Corriden R, Insel PA (2010). Basal release of ATP: an autocrine-paracrine mechanism for cell regulation.. Sci Signal.

[18] Correa-Meyer E, Pesce L, Guerrero C, Sznajder JI (2002). Cyclic stretch activates ERK1/2 via G proteins and EGFR in alveolar epithelial cells.. Am J Physiol Lung Cell Mol Physiol.

[19] Togo T, Alderton JM, Steinhardt RA (2003). Long-term potentiation of exocytosis and cell membrane repair in fibroblasts.. Mol Biol Cell.

[20] Burnstock G (2006). Pathophysiology and therapeutic potential of purinergic signaling.. Pharmacol Rev.

[21] Abbracchio MP, Burnstock G, Boeynaems JM, Barnard EA, Boyer JL (2006). International Union of Pharmacology LVIII: update on the P2Y G protein-coupled nucleotide receptors: from molecular mechanisms and pathophysiology to therapy.. Pharmacol Rev.

[22] dos Santos CC, Han B, Andrade CF, Bai X, Uhlig S (2004). DNA microarray analysis of gene expression in alveolar epithelial cells in response to TNFalpha, LPS, and cyclic stretch.. Physiol Genomics.

[23] Pugin J (2003). Molecular mechanisms of lung cell activation induced by cyclic stretch.. Crit Care Med.

[24] da Cruz CM, Ventura AL, Schachter J, Costa-Junior HM, da Silva Souza HA (2006). Activation of ERK1/2 by extracellular nucleotides in macrophages is mediated by multiple P2 receptors independently of P2X7-associated pore or channel formation.. Br J Pharmacol.

[25] Ralevic V, Burnstock G (1998). Receptors for purines and pyrimidines.. Pharmacol Rev.

[26] Wesley UV, Bove PF, Hristova M, McCarthy S, van der Vliet A (2007). Airway epithelial cell migration and wound repair by ATP-mediated activation of dual oxidase 1.. J Biol Chem.

[27] Reddy A, Caler EV, Andrews NW (2001). Plasma membrane repair is mediated by Ca(2+)-regulated exocytosis of lysosomes.. Cell.

[28] Ware LB, Matthay MA (2000). The acute respiratory distress syndrome.. N Engl J Med.

[29] Halbertsma FJ, Vaneker M, Scheffer GJ, van der Hoeven JG (2005). Cytokines and biotrauma in ventilator-induced lung injury: a critical review of the literature.. Neth J Med.

[30] dos Santos CC, Slutsky AS (2006). The contribution of biophysical lung injury to the development of biotrauma.. Annu Rev Physiol.

[31] Covian-Nares JF, Koushik SV, Puhl HL, Vogel SS (2010). Membrane wounding triggers ATP release and dysferlin-mediated intercellular calcium signaling.. J Cell Sci.

[32] Wang EC, Lee JM, Ruiz WG, Balestreire EM, von Bodungen M (2005). ATP and purinergic receptor-dependent membrane traffic in bladder umbrella cells.. J Clin Invest.

[33] Li J, Liu D, Ke HZ, Duncan RL, Turner CH (2005). The P2X7 nucleotide receptor mediates skeletal mechanotransduction.. J Biol Chem.

[34] Liu D, Genetos DC, Shao Y, Geist DJ, Li J (2008). Activation of extracellular-signal regulated kinase (ERK1/2) by fluid shear is Ca(2+)- and ATP-dependent in MC3T3-E1 osteoblasts.. Bone.

[35] Kiefmann R, Islam MN, Lindert J, Parthasarathi K, Bhattacharya J (2009). Paracrine purinergic signaling determines lung endothelial nitric oxide production.. Am J Physiol Lung Cell Mol Physiol.

[36] Rossi AH, Salmon WC, Chua M, Davis CW (2007). Calcium signaling in human airway goblet cells following purinergic activation.. Am J Physiol Lung Cell Mol Physiol.

[37] Adriaensen D, Timmermans JP (2004). Purinergic signalling in the lung: important in asthma and COPD?. Curr Opin Pharmacol.

[38] Goding JW (2000). Ecto-enzymes: physiology meets pathology.. J Leukoc Biol.

[39] Homolya L, Steinberg TH, Boucher RC (2000). Cell to cell communication in response to mechanical stress via bilateral release of ATP and UTP in polarized epithelia.. J Cell Biol.

[40] Rice WR, Burton FM, Fiedeldey DT (1995). Cloning and expression of the alveolar type II cell P2u-purinergic receptor.. Am J Respir Cell Mol Biol.

[41] Gozal E, Forman HJ, Torres M (2001). ADP stimulates the respiratory burst without activation of ERK and AKT in rat alveolar macrophages.. Free Radic Biol Med.

[42] Vlahakis NE, Schroeder MA, Pagano RE, Hubmayr RD (2001). Deformation-induced lipid trafficking in alveolar epithelial cells.. Am J Physiol Lung Cell Mol Physiol.

[43] Vlahakis NE, Schroeder MA, Pagano RE, Hubmayr RD (2002). Role of deformation-induced lipid trafficking in the prevention of plasma membrane stress failure.. Am J Respir Crit Care Med.

[44] Zhang Z, Chen G, Zhou W, Song A, Xu T (2007). Regulated ATP release from astrocytes through lysosome exocytosis.. Nat Cell Biol.

[45] Beigi RD, Dubyak GR (2000). Endotoxin activation of macrophages does not induce ATP release and autocrine stimulation of P2 nucleotide receptors.. J Immunol.

[46] Lazarowski ER, Harden TK (1999). Quantitation of extracellular UTP using a sensitive enzymatic assay.. Br J Pharmacol.

[47] Lazarowski ER, Boucher RC, Harden TK (2000). Constitutive release of ATP and evidence for major contribution of ecto-nucleotide pyrophosphatase and nucleoside diphosphokinase to extracellular nucleotide concentrations.. J Biol Chem.

[48] McNeil PL (2002). Repairing a torn cell surface: make way, lysosomes to the rescue.. J Cell Sci.

[49] Terasaki M, Miyake K, McNeil PL (1997). Large plasma membrane disruptions are rapidly resealed by Ca2+-dependent vesicle-vesicle fusion events.. J Cell Biol.

[50] Liu GJ, Werry EL, Bennett MR (2005). Secretion of ATP from Schwann cells in response to uridine triphosphate.. Eur J Neurosci.

[51] Sugamoto Y, Hirai K, Tokoro T (1999). P2Y2 receptor elevates intracellular calcium concentration in rabbit eye suprachoroid.. J Med Dent Sci.

[52] Seino S, Shibasaki T (2005). PKA-dependent and PKA-independent pathways for cAMP-regulated exocytosis.. Physiol Rev.

[53] Orihuela PA, Parada-Bustamante A, Zuniga LM, Croxatto HB (2006). Inositol triphosphate participates in an oestradiol nongenomic signalling pathway involved in accelerated oviductal transport in cycling rats.. J Endocrinol.

[54] Schrader AM, Camden JM, Weisman GA (2005). P2Y2 nucleotide receptor up-regulation in submandibular gland cells from the NOD.B10 mouse model of Sjogren's syndrome.. Arch Oral Biol.

[55] Leonard RT, Hotchkiss CW (1978). Plasma Membrane-associated Adenosine Triphosphatase Activity of Isolated Cortex and Stele from Corn Roots.. Plant Physiol.

[56] Oeckler RA, Hubmayr RD (2007). Alveolar microstrain and the dark side of the lung.. Crit Care.

[57] Tschumperlin DJ, Margulies SS (1999). Alveolar epithelial surface area-volume relationship in isolated rat lungs.. J Appl Physiol.

[58] Chang SJ, Tzeng CR, Lee YH, Tai CJ (2008). Extracellular ATP activates the PLC/PKC/ERK signaling pathway through the P2Y2 purinergic receptor leading to the induction of early growth response 1 expression and the inhibition of viability in human endometrial stromal cells.. Cell Signal.

[59] Takai Y, Sasaki T, Matozaki T (2001). Small GTP-binding proteins.. Physiol Rev.

[60] Okada SF, Nicholas RA, Kreda SM, Lazarowski ER, Boucher RC (2006). Physiological regulation of ATP release at the apical surface of human airway epithelia.. J Biol Chem.

[61] Skaper SD, Debetto P, Giusti P (2010). The P2X7 purinergic receptor: from physiology to neurological disorders.. Faseb J.

[62] Weisman GA, Wang M, Kong Q, Chorna NE, Neary JT (2005). Molecular determinants of P2Y2 nucleotide receptor function: implications for proliferative and inflammatory pathways in astrocytes.. Mol Neurobiol.

[63] Baker OJ, Camden JM, Rome DE, Seye CI, Weisman GA (2008). P2Y2 nucleotide receptor activation up-regulates vascular cell adhesion molecule-1 [corrected] expression and enhances lymphocyte adherence to a human submandibular gland cell line.. Mol Immunol.

[64] Boitano S, Safdar Z, Welsh DG, Bhattacharya J, Koval M (2004). Cell-cell interactions in regulating lung function.. Am J Physiol Lung Cell Mol Physiol.

[65] Matsuyama H, Amaya F, Hashimoto S, Ueno H, Beppu S (2008). Acute lung inflammation and ventilator-induced lung injury caused by ATP via the P2Y receptors: an experimental study.. Respir Res.

[66] Kolosova IA, Mirzapoiazova T, Moreno-Vinasco L, Sammani S, Garcia JG (2008). Protective effect of purinergic agonist ATPgammaS against acute lung injury.. Am J Physiol Lung Cell Mol Physiol.

[67] Wang S, Hubmayr RD (2011). Type I Alveolar Epithelial Phenotype in Primary Culture..

[68] Stroetz RW, Vlahakis NE, Walters BJ, Schroeder MA, Hubmayr RD (2001). Validation of a new live cell strain system: characterization of plasma membrane stress failure.. J Appl Physiol.

[69] Belete HA, Godin LM, Stroetz RW, Hubmayr RD (2010). Experimental models to study cell wounding and repair.. Cell Physiol Biochem.

[70] Green AS (2004). Modelling of peak-flow wall shear stress in major airways of the lung.. J Biomech.

